# Interdisciplinary collaboration in serious illness conversations in patients with multiple myeloma and caregivers – a qualitative study

**DOI:** 10.1186/s12904-023-01221-5

**Published:** 2023-07-13

**Authors:** Cæcilie Borregaard Myrhøj, Dorte Toudal Viftrup, Mary Jarden, Stine Novrup Clemmensen

**Affiliations:** 1 Department of Hematology, Copenhagen University Hospital, Blegdamsvej 9, Rigshospitalet, Copenhagen, 2100 Denmark; 2 Cancer Survivorship and Treatment Late Effects (CASTLE), Department of Oncology, Copenhagen University, Blegdamsvej 58, Rigshospitalet, Copenhagen, 2100 Denmark; 3 Department of Public Health, Research Unit of General Practice, University of Southern Denmark, Odense C, 5000 Denmark; 4 Department of Clinical Medicine, University of Copenhagen, Blegdamsvej 3B, Copenhagen, 2200 Denmark

**Keywords:** Interdisciplinary Communication, Cancer, Critical illness, Professional-Patient Relations, Multiple myeloma, Qualitative research

## Abstract

**Background:**

There is growing evidence that conversations between healthcare professionals and patients with serious illness can improve the quality of end-of-life cancer care. Yet, there is lack of insight into how different healthcare professions collaborate to deliver serious illness communication, as well as patients’ and caregivers’ perceptions of this collaboration between the nurse and physician. This study explores the interdisciplinary collaboration between nurses and physicians in serious illness conversations with patients diagnosed with multiple myeloma and their caregivers.

**Methods:**

Eleven dyadic interviews were conducted with 22 patients and caregivers, and two focus group interviews involving four nurses and the other with four physicians. Data analysis and reporting were conducted using reflexive thematic analysis within phenomenological epistemology.

**Results:**

The interdisciplinary collaboration was characterized by three main themes: (1) Importance of relationships, (2) Complementary perspectives, and (3) The common goal.

**Conclusion:**

This study highlights the importance of interdisciplinarity in serious illness conversations as it enhances the use of existential and descriptive language when addressing medical, holistic, and existential issues. The use of broader language also reflects that interdisciplinary interaction strengthens the expertise of each professional involved in patient care. Through interdisciplinary collaboration, the preferences, hopes, and values of the patient and caregiver can be integrated into the treatment plan, which is key in providing the delivery of optimal care. To promote cohesive and coordinated collaboration, organizational changes are recommended such as supporting continuity in patient–healthcare professional relationships, providing interdisciplinary training, and allocating time for pre-conversation preparation and post-conversation debriefing.

## Background

Hematological malignancies differ widely in severity and prognosis. Multiple Myeloma (MM) is an incurable hematological malignancy with a 5-year survival rate of approximately 60% for patients in the Nordic countries [1]. Patients with MM have a substantial symptom burden with acute and chronic psychosocial and medical needs which require engagement of the interdisciplinary team [2–4]. Due to poor survival and high symptom burden in patients with MM, preparing patients and caregivers for serious illness and end-of-life issues should be part of interdisciplinary MM cancer care [5]. Research supports the recommendation of an interdisciplinary team approach for engaging in serious illness and end-of-life conversations. However, studies also reveal a degree of uncertainty about role responsibilities within the interdisciplinary team [6, 7].

Several studies point to the positive association between interdisciplinary collaboration in patient conversations and the provision of high quality and valuable healthcare [6, 8–11]. Evidence shows that adapting patient care and treatment plans to align with the patient’s individual goals lead to improved quality of end of life treatment [8, 9, 11–13]. However, there is limited knowledge regarding serious illness communication within Hematology, and only a few studies report successful implementation of serious illness conversations in this context [14, 15]. A multi-component concept called The Serious Illness Care Program (SICP) [16] has focused on strengthening communication in serious illness conversations, resulting in a significant increase in the frequency and timing of these conversations, while adapting an enhanced person-centered approach [17]. We used the UK Medical Research Council’s framework for complex interventions [18] to adapt and develop the ‘serious illness conversation’ concept for MM patients. The adaptation process involved collaboration with four hematological nurses, four hematology specialists, six MM patients and four caregivers. Details of the development and evaluation have been reported in a submitted article. To refine the adapted concept, we conducted a pilot test of the Danish version, involving patients with MM and their caregivers during a five-month period. A recent qualitative study explored the experiences of serious illness conversations among patients with MM, and how aspects of being seriously ill were discussed in these conversations, but did not report on aspects of interdisciplinary collaboration during these conversations [5].

### Interdisciplinary collaboration

Interdisciplinary collaboration refers to the process where individuals from different health professions work together to positively impact patient care [19]. This collaborative approach integrates the unique skills and expertise of each professional through negotiated interaction, contributing to comprehensive patient care [7, 20]. However, studies of interdisciplinary collaboration have shown it can be fragmented and uncoordinated due to challenges such as imbalance of authority, limited understanding of other’s roles and responsibilities [21, 22]. Lakin et al. investigated the interdisciplinary collaboration within SICP in primary care and recommended strategies to improve serious illness conversations conducted by interdisciplinary teams. These strategies include defining clear roles for professionals and emphasizing interdisciplinary and clinician-patient relationships [7]. However, to our knowledge, there are no studies exploring the experiences of nurses and physicians in interdisciplinary collaboration when engaging patients with hematological cancers, such as MM, in serious illness communication or how patients and caregivers experience this interdisciplinary collaboration.

The aim of this study was to explore the experiences and perceptions of patients, caregivers, physicians, and nurses regarding interdisciplinary collaboration between nurses and physicians during serious illness conversations. Further, to understand the impact of specific roles within the collaboration on communication during serious illness conversations.

## Method

### Serious illness conversations in hematology – an adapted concept

Serious illness conversation is centered around the patient’s current experience of illness emphasizing the importance of establishing a secure environment for the patient to openly express their perspectives and preferences to a HCP who understands the disease and medical aspects [23]. The serious illness conversation is a structured intervention in which HCPs provide patients with information about their illness and prognosis in accordance with their preferences. During the conversation, the HCP explores the goals, values, and priorities of the patient and caregiver, and provides a medical recommendation for next steps in care. Importantly, the serious illness conversation can be revisited throughout the illness trajectory [23].

The Danish adaptation of the serious illness conversation concept involves interdisciplinary collaboration requiring the participation of both a nurse and physician. The interdisciplinary team of nurses and physicians undergo an 8-hour training session to prepare for conducting these conversations. During the training session, nurses and physicians are trained to actively engage in the conversation, through either a dialogue or a more sentient role, and to transition between these two roles as needed. The training also emphasizes structuring the conversation and fostering mutual support skills between nurses and physicians. Additionally, the adapted conversation is “unhurried”, with one hour allocated for both interdisciplinary collaboration dialogues before and after the conversation, the conversation itself and the subsequent documentation. Identification of specific timepoints for inviting patients and caregivers for a conversation was done in collaboration with HCP’s and patients and caregivers at a mutual workshop. The following timepoints for MM patients were chosen: A few weeks after time of diagnosis, at time of the first relapse, at each relapse after 3rd line of treatment, and at transition to palliative care.

Patients and caregivers receive preparatory materials that facilitate prior reflection on topics as prognosis, quality of life, and existential issues, helping them identify the specific issues they wish to address during the conversation [5].

### Research design

This qualitative study, grounded in phenomenological epistemology, explored the experiences and perceptions of interdisciplinary collaboration during serious illness conversations from the perspective of patients, caregivers, nurses, and physicians. We carried out 11 dyadic interviews with patients and caregivers, and two focus group interviews involving nurses and physicians, respectively. Both dyadic and focus group interviews were congruent as methods to the research question. Dyadic and focus group interviews have the advantage of enrichment of data in a manner that is not possible in individual interviews [24, 25]. An interaction between several participants can contribute to the phenomenon being elucidated from several perspectives. This approach allows participants to listen to each other’s descriptions, reflect on them, and then contribute with their own perspectives and experiences [24, 25].

Data analysis was conducted using Reflexive Thematic Analysis [26], a theoretically flexible approach suitable for analyzing qualitative data across a range of theoretical and epistemological perspectives [26, 27]. In this study, it was applied within phenomenological epistemology. This method was considered appropriate given the combination of focus group interviews and dyadic interviews conducted in this study.

### Participants, recruitment, and sampling procedures

Participants were recruited from the Department of Hematology, Copenhagen University Hospital, Rigshospitalet, between October 2019 and January 2020 and included in the pilot study testing the concept of Serious Illness Conversations in patients with MM and their caregivers.

Patients diagnosed with MM and their caregivers were eligible for inclusion if they were ≥ 18 years, and able to read and speak Danish. They were required to have participated in at least one serious illness conversation. Participants with unstable mental disorders or psychiatric diagnoses were excluded from the study. During the recruitment period, a total of 14 serious illness conversations were conducted with patients and caregivers. Recruitment of patients (n = 11) and their caregiver (n = 11) took place on the same day as the conversation. Patients were male (n = 7) and female (n = 4), aged 54–89 years (mean age = 69), with educational backgrounds ranging from no higher education (n = 3), short higher education < 3 years (n = 3), to medium higher education = 3–4 years (n = 6), and were receiving first-line treatment (n = 6), second-line treatment (n = 1), third- line treatment (n = 1), fourth-line treatment or more (n = 3). Caregivers were male (N = 3) and female (n = 8), aged 23–85 years (mean = 63), with educational degree in the range from no higher education (n = 4), short higher education < 3 years (n = 2), medium higher education = 3–4 years (n = 4), to long higher education > 5 years (n = 1). One patient and caregiver failed to meet the inclusion criteria due to dementia, and one patient and caregiver accepted participation but were unable to participate due to medically unstable disease. One patient attended the conversation without a caregiver.

HCPs were male (n = 1) and female (n = 7), aged 38–59 years (mean = 49), either a nurse (n = 4) or physician (n = 4), had > 2 years of experience working with patients with MM (mean 15, range 4–30), had participated in an interdisciplinary training session, and completed at least one serious illness conversation in patients with MM and their caregiver (mean 5, range 1–10).

A purposive and pragmatic sampling was applied to ensure a diverse study sample across gender, life situation, working experience, and relationship of caregivers. All participants were approached by the first author and informed consent was obtained prior to conducting the interviews. All participants were interviewed within three weeks of their inclusion in the study. Although the first author was a colleague of the HCPs, there was no formal personnel management responsibilities or existing relations to patients and caregivers. Participants were informed that they had the option to withdraw from the study at any time without any consequences to their medical care.

The sample size and information power of the study were considered appropriate, taking into account the research aim, sample specificity of the sample, quality of dialogue, and analysis strategy [28].

### Data collection

The interviews were conducted by the first author, a clinical nurse specialist, and the second author, a trained psychologist. Both had been involved in training the HCPs prior to the conversations, but they had no prior relationship with the patients or caregivers. During the interviews, participants were encouraged to provide their own descriptions, and follow-up questions using ‘what’ and ‘how’ to gain a more comprehensive understanding of their experiences and perceptions of the phenomenon. The length of the dyadic interviews ranged between 34 and 67 min (mean 55) and the two focus group interviews were 73 and 76 min.

#### Dyadic interviews

Eleven semi-structured dyadic interviews were conducted with patients and caregivers, within a mean of eight days (range: 2–20 days) following the conversation. The interviews were conducted at the participants’ preferred location; either at their private home (n = 1) or a private room at the hospital (n = 10). The interview guide focused on exploring participants’ experiences of the dynamics between the physician and nurse during the serious illness conversation (Table 1).

**Table 1:** Interview guide for dyad interviews

Topics	Research questions	Interview questions
**Interdisciplinary Involvement in the conversation**	How do patients and caregivers perceive the dynamics of the conversation between physician, nurse, caregiver, and patient?	- How did you feel while participating in the conversation? - What was your experience of the physician during the conversation? - What was your experience of the nurse during the conversation?
**New setting**	What is the patient’s and caregiver’s experience of having both the physician and nurse present during the conversation?	- How did the presence of both a physician and a nurse effect the dynamics and content of the conversation?
**Individual perspectives**	Do patients and caregivers feel that their perspectives are adequately considered and included by both the nurse and physician during the conversation?	- What was the main focus of the conversation as you experienced it? How was it emphasized or directed, and by whom (physician, nurse or both?

#### Focus group interviews

The two focus group interviews were conducted after five-month pilot testing of the concept. The focus group interviews were conducted in undisturbed rooms at the hospital. The first focus group interview included nurses, and the second focus group interview involved physicians. Separate interviews were conducted with each profession to ensure a free and safe atmosphere, as it was uncertain how their interactions in the interview might be influenced by their daily clinic interactions.

The interview guide centered on how HCPs experienced the collaboration between nurses and physicians in relation to interdisciplinarity, shared professionalism, and their professional roles during the conversation (Table 2).

**Table 2:** Interview guide for focus group interview

Topics	Research questions	Questions and preliminaries
**Introduction**	Set the scene for the focus group interview. Information about Interaction.	This focus group interview will be conducted as an interactive conversation between the participants. We want to learn from your experiences during your conversations. Your thoughts, experiences, and reflections are important to us, and there are no wrong answers. By sharing your perspectives, you contribute to the co-creation of learning and knowledge about these conversations. As the facilitator, my role is to ask questions and ensure that everyone has the opportunity to contribute I will also manage the time and guide the discussion to cover relevant topics that need to be addressed.
**Involvement**	How did the nurses and physicians experience the interdisciplinary collaboration during the conversations? How did they perceive the dynamics of the conversation? What is the significance or importance of this collaboration?	Individual reflection questions. Participants are provided with a sheet of paper to write on. When everyone is done writing we will open for discussion: - How was the interdisciplinary collaboration expressed during the conversation? - What do you perceive as the roles of the nurse and the physician respectively during the conversation? - Discuss the individual roles and contributions of each participant.
**Round-off**	How does the nurse/physician experience the significance of these interdisciplinary serious illness conversations?	How do you, as a physician/nurse, experience the benefit of these interdisciplinary conversations?

### Data analysis

The interviews were audio-recorded and transcribed verbatim. Reflexive Thematic Analysis by Braun and Clark [27] was used to identify themes and patterns of meaning across the dataset [26]. The analysis process involved six steps, with all authors contributing with their different theoretical perspectives from nursing, medical, and psychological backgrounds to enhance the understanding and interpretation of the data. In step one, the first and second author became familiar with the data by reading and re-reading the interview transcripts to gain a comprehensive understanding. Initial impressions were noted. In step two, the data were systematically organized by dividing the text into meaning units. Descriptions and meaning units were transformed into concise phrases focusing on the interdisciplinary collaboration from a nursing perspective. Initial codes were generated and discussed within a nursing, psychology, and medical perspective by three authors (first, second, and last). New codes were generated and existing ones were refined. In step three, the initial codes were examined for each description and transformed into overarching themes across all descriptions, following an idiographic approach. In step four, all authors reviewed, modified, and refined the preliminary themes, ensuring their coherence within the entire data set, and across both the dyadic and focus group interviews. In step five, the essence of the themes and their relationships to each other and to identified subthemes were identified [27]. Finally, in step six, the final analysis and results were compiled and drafted. See Table 3 for an example of step one to five and the involvement of authors throughout the analysis process.

**Table 3:** Thematic analysis process – example (Involvement of authors throughout the analysis process)

Step 1 (First and second author)	Step 2 (First author)	Step 3 (First, second and last author)	Step 4 (All authors)	Step 5 (All authors)
Quotation	**Code and interpretation**	**Potential theme**	**Identified subtheme**	**Overarching theme**
Nurse 4: “*even though I didn’t know the patient and caregiver, I think my presence alone gave the caregiver the courage to get more involved in the conversation.”* Caregiver 8: “*it was a different conversation when the nurse was there, she asked personal questions rather than the typical medical inquiries – more about us as individuals.”* Physician 2: “*I know a lot about the treatment and the disease, but the nurses often possess a deeper, more personal understanding of the patient.”* Patient 5: *“The conversation becomes more relaxed when we already know the physician and nurse, and they know us.”*	Contribution by being present Different questions Different knowledge ´ Prior knowledge	Caregiver involvement Nursing impact Be seen as a person Medical vs. personal knowledge Prior relationship Trust	Contribution	Importance of relationships
Patient 11: “*it didn’t feel like it was a nurse and a physician in front of us. It felt more like two people that were interested in getting to know me better”* Physician 3 *”As a physician, my purpose with this conversation is to align the medical care plan with the wishes and preferences of the patient.”* Patient 3: “*She [the nurse] helped us to understand the physician’s explanations about the treatment and how it would affect our everyday life.”*	No clear division of roles Relationship defines the role	Merging roles Medical role Nursing role	Roles	Importance of relationships

## Findings

**Fig. 1:**
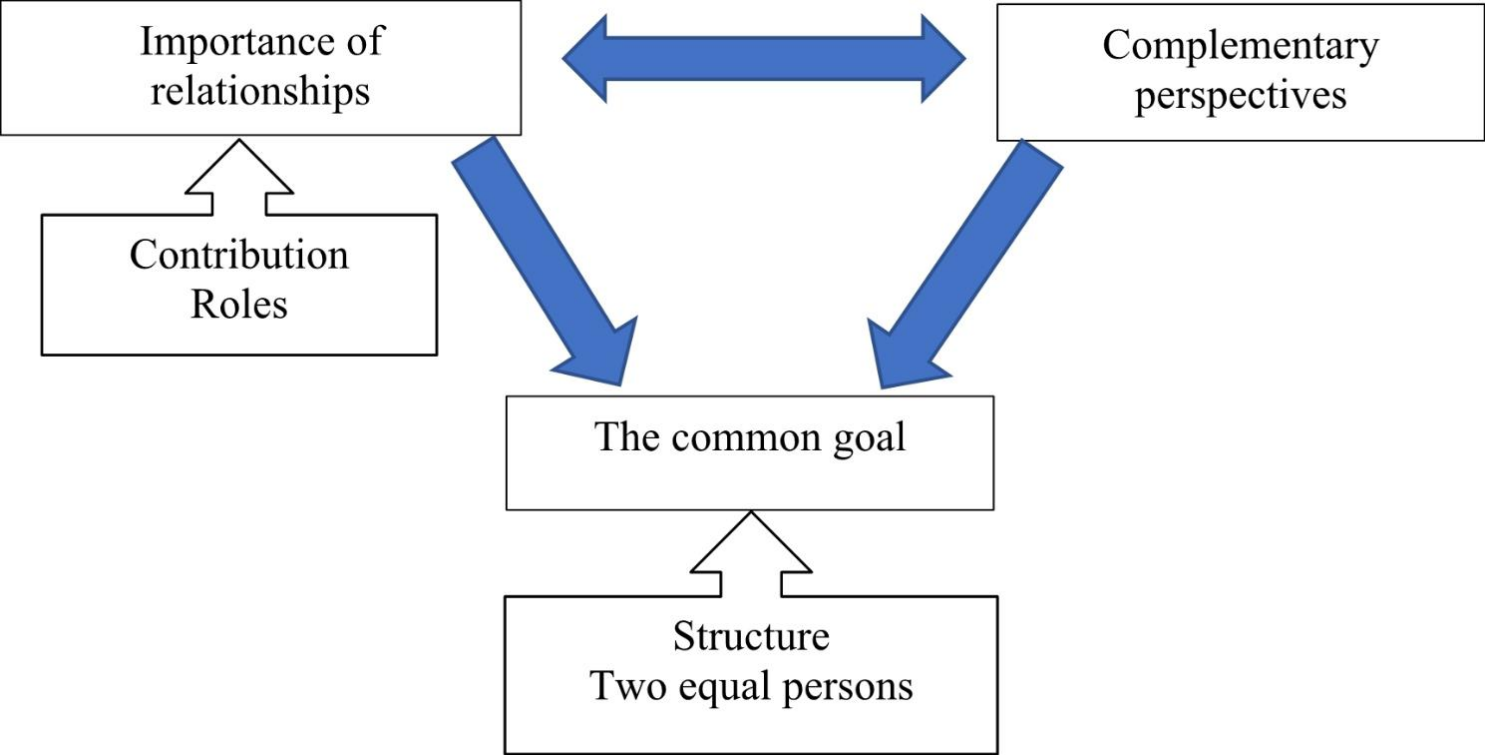
The characteristics of the interdisciplinary collaboration in serious illness conversations *The prior relationship between healthcare professionals and patients influence their respective contributions to the interdisciplinary collaboration and the extent to which their (nurse’s or physician’s) different perspectives play an important role in the conversation. Concurrently, the relationship itself is also affected by these perspectives, thereby contributing to the interdisciplinary collaboration. Both perspectives and relationships have an impact on the common goal*

The interdisciplinary collaboration between nurses and physicians during serious illness conversations was characterized by three themes:: “Importance of relationships”, “Complementary perspectives,” and “The common goal”. The general structure of the interdisciplinary collaboration and the interrelationship between the three themes are illustrated in Fig. 1. In the following section, we will provide detailed explanations and expand upon each identified theme.

### Theme 1: importance of relationships

The prior relationship between HCPs (nurses and physicians) and patient and caregiver had a significant influence on interdisciplinary collaboration. This relationship directly affected the contributions and roles of each profession during the conversations.

#### The contribution

Several nurses and physicians experienced that establishing a better relationship with the patient led to a greater contribution within the interdisciplinary collaboration. The HCP who had the most knowledge of the patient’s situation, coupled with a strong patient relationship, took charge of the conversation, with other HCPs following their lead.

*“I’ve had two conversations without having a prior relationship with the patient, but the physician was well acquainted with them. While the conversation was okay, I didn’t feel that I contributed a lot. The best conversations are the ones where we both know the patient really well.” (Nurse 3).*

Additionally, patients and caregivers reported a noticeable improvement in communication with the healthcare team (nurse and physician) when a prior relationship had been established. This familiarity allowed for more personalized conversations that were centered around the patients’ values and preferences.

*”It was nice to have the same physician as during the treatment. She was familiar with our background, so we could just pick up the conversation from last time. We didn’t have to start from scratch and explain everything, as she [the physician] already had the necessary knowledge about us.” (Patient 2)*

#### The roles

Nurses experienced taking a more sentient role in the interdisciplinary collaboration when they did not have a prior relationship with the patient. Leading in the conversation and asking questions became more difficult in such cases. However, when the nurses had an established relationship with the patient and caregiver, their role became more active, fostering an open dialogue that facilitated easier discussions of existential matters and inquires. In addition, the nurse’s role was to maintain the conversation’s focus on what was important to the patient and caregiver, rather than allowing it to become a routine medical consultation.

*“When the physician knows the patient best, the conversation quickly turns toward blood test results and medical matters. But as a nurse, if I also know the patient well, we often get back to some of the things they truly want to talk about, like the more existential issues, right?” (Nurse 3)*

The interdisciplinary collaboration was experienced by patients and caregivers as more natural and relaxed when they had an established relationship with both the nurse and physician prior to the conversation. Furthermore, this familiarity fostered a sense of equal partnership between the nurse and physician, with both perspectives making valuable contributions to the conversation. However, the relationship with the patient and caregiver was influenced by the HCPs profession, given that their daily tasks and responsibilities in the patient’s disease trajectory were defined by their respective professions. The physician was responsible for devising the treatment plan, while the nurse primarily focused on practical aspects such as administering treatment and identifying the needs of the patient and caregiver needs.

“O*ne of us focuses on the treatment, while the other takes care of the practicalities of everyday life, but we are united about the future.” (Nurse 4)*

The role of the physician was predominantly defined by their responsibility for devising the patient’s medical care plan, and this also influenced their relationship with the patient.

*” As a physician, my purpose with this conversation is to align the medical care plan with the wishes and preferences of the patient.” (Physician 3)*

The HCPs’ relationship with patients and caregivers and their different roles and responsibilities in clinical practice, influenced the outcome of the conversations. When both the nursing and physician roles were active, the consultation was perceived as being more patient-centered, incorporating discussions related to treatment as well as everyday life issues.

### Theme 2: complementary perspectives

The interdisciplinary collaboration was described by patients, caregivers, physicians, and nurses as being complementary. The complementarity was composed from the two different perspectives of the nurse and the physician during the conversation. The nursing perspective entailed a heightened awareness of emotions, emphasizing the importance of naming, supporting, and acknowledging the emotional reactions of patients and caregivers. In this way, nurses effectively communicated unspoken observations in the room.

*“I help to articulate the emotions or reactions I observe, because the physician focuses on explaining the disease trajectory.” (Nurse 2)*

Nurses frequently developed a more personal relationship with the patient and caregiver, enabling them to naturally emphasize the values important to patients and caregivers. This complemented the perspective of the physician, whose relationship with the patient was defined by their medical knowledge and responsibility for the treatment.

*” The physician may hear one thing and focus on it, while the nurse might overhear something that they are accustomed to paying attention to. (…) I think that having both aspects represented in the conversations lead to a higher quality outcome.”(Physician 1)*

The physicians brought their specialized knowledge and comprehensive understanding of the disease trajectory, prognosis, and future medical treatment options into the conversation. In contrast, the nurses acknowledged that they did not have the same level of overview or expertise to discuss prognosis and treatment plans. Instead, they took the opportunity to incorporate the perspectives of the patient and caregiver when discussing prognosis and medical treatment.

*“The physicians have an overview of the treatment trajectory, which I don’t have. And this overview brings a sense of calm to the patient and caregiver during the conversation, allowing me to focus on helping them to express what matters to them.” (Nurse 3)*

All patients and caregivers recognized the complementary nature of the nursing perspective in relation to the physician’s role, which enhanced their understanding of the physician’s communication. The nurses played an important role in ‘translating’ the medical terminology into everyday language and explaining how treatments and procedures impact their daily lives.

*“In the majority of the conversations, the physician asked me to explain certain practical matters that they couldn’t address themselves, such as how to obtain support for daily life. So, in this way, both of us contributed to clarifying different aspects during the conversation.” (Nurse 1)*

a result, these conversations led to improved collaboration between physicians and nurses in their daily clinical practice, as they recognized the value of complementing each other’s roles. This resulted in greater satisfaction in the care provided to patients and caregivers.

The majority of patients and caregivers shared the experience that both the physician and nurse brought different perspectives and inquiries to the conversation, making them complementary to each other. The alignment of these perspectives towards a common goal fostered a collaborative approach that benefited the overall care process.

### Theme 3: the common goal

The nurse and physician worked collaboratively as equals, following a unified and structured approach during the conversation, with the common objective of providing the best possible care for the patient and caregiver.

#### Structure

The nurses and physicians frequently engaged in pre- conversation and post-conversation meetings for preparation and debriefing purposes. These interdisciplinary discussions were of importance, particularly for the nurses.

*“It means a lot to have the opportunity to prepare together. The physician can provide a brief overview of the patient’s disease trajectory and treatment plan, and highlight the topics they expect will be brought up during the conversation.” (Nurse 2)*

The pre-meeting discussions played an important role in establishing a collaborative structure for the conversations.

*” The preparation time allows us to collectively figure out the direction [of the conversation] and identify what is important to focus on from our respective perspectives.” (Nurse 4)*

The interdisciplinary nature of the conversations ensured that the common goal remained the main focus. Patients and caregivers expressed appreciation for the nurse’s role in facilitating the conversations, by maintaining structure and purpose. This involved setting the agenda, summarizing key points, and following up as needed. The collaborative and structured approach, both prior to and during the conversations, helped to sustain the focus on the shared goal of the conversation.

#### Two equal persons

The interdisciplinary collaboration was perceived as a partnership between equals, with neither the medical nor nursing profession dominating the conversation. Both nurses and physicians shared a mutual goal of developing a unified plan for the benefit of the patient and caregiver.

*” In this conversation we both [nurse and physician] agree to help this person, who needs to be taken care of, and then you get this sense of connection with another colleague.” (Physician 2)*

Interdisciplinarity during the conversations was highlighted by both nurses and physicians as providing great benefit and support for maintaining focus in the conversation.

*“Well, I think it is nice there are two of us in control of the conversation. If you are suddenly stuck in one particular topic or focus, the other could redirect the conversation towards what matters to the patient and caregiver.”(Physician 4)*

The presence of both the physician and the nurse in the conversation created a distinct context, contrasting with a typical medical consultation. Patients and caregivers described a more relaxed and natural setting, with less emphasis on medical treatment and more attention given to their specific needs and concerns

*“The presence of the nurse changes the dynamics and differs from the normal setting [medical consultation]. It feels like there is a group gathered together, talking to each other. It feels like a different kind of interaction and consultation.” (Caregiver 10)*.

The nurse being present in the conversation brought about a shift in topics and agenda, resulting in an interdisciplinary collaboration that integrated medical concerns with existential issues relevant to the patients and their caregiver’s everyday life. Patients, caregivers, nurses and physicians recognizes this collaboration as a unifying force that addresses the holistic needs of the patient and caregiver.

## Discussion

The study aimed to explore patients’, caregivers’, physicians’, and nurses’ experiences of interdisciplinary collaboration between nurses and physicians during serious illness conversations, and how their roles affected communication during these conversations.

### Statement of principal findings

The findings of this study provide novel insights into the experiences of patients, caregivers, physicians, and nurses regarding the interdisciplinary collaboration between nurses and physicians during serious illness conversations. The study highlights the significance of prior relationships with patients and caregivers, which influences the contribution and roles within the interdisciplinary collaboration, particularly for nurses. The nurse and physician bring different perspectives based on their knowledge and relationships with the patient and the caregiver, and these perspectives complement each other, fostering effective interdisciplinary collaboration. Both the relationships and the perspectives of the nurse and physician were aligned towards the shared goal of providing the best care for the patients and caregivers.

To the best of our knowledge, this is the first study to explore the interdisciplinary collaboration between nurses and physicians during serious illness conversations, from the perspective of patients, caregivers, physicians, and nurses involved in the conversations.

### Findings in relations to other studies

We found that the HCP’s prior relationship with the patient had a notable impact on their participation in interdisciplinary collaboration. Nurses tended to assume a more reserved role when they had no prior relationship with the patient but became more involved and active in the conversations when they were already acquainted with the patient. Similarly, an integrative review of 15 articles found that nurses who had established relationships with patients were more supportive and engaged during end-of-life conversations [29]. Our study found that nurses lacked a comprehensive overview of the individual treatment of the patients, which is consistent with the findings of Ikander et al. However, nurses described their role as translating medical terms into everyday language to enhance patient understanding, while physicians provided medical overviews and prognostic information. In contrast to Ikander et al., our study found clear role definitions even when the conversation focused on medical aspects [29]. HCPs in our study underwent joint training as a team prior to the conversations, which likely prompted the establishment of clear nursing roles. Our findings of clear professional roles and responsibilities during the conversation, as well as the importance of the clinician-patient relationship are highlighted to improve the conversations was also emphasized by Lakin et al. [7]. These findings support the idea that prior relationships, role clarification and team training contribute to effective interdisciplinary collaboration and enhance the quality of conversations between HCPs and patients.

Our study showed that nurses offered a more personal understanding of the patient and caregiver, providing a distinct perspective compared to physicians who approached the patient primarily from a medical standpoint based on their knowledge and responsibility for medical treatment. Despite these differing perspectives, all participants in the conversation experienced the interdisciplinary collaboration as a collaboration between two equals, driven by a common goal. In contrast, previous studies have reported that physicians may create barriers to effective collaboration by failing to recognize the importance of the nurses’ professional role in communication [30], and perceiving interdisciplinary collaboration as less important [31]. Nevertheless, Lakin et al. found that when the nurse and physician perspectives integrate they strengthen the output of the conversation [7], which is also in line with the findings of this study. Our study also found that interdisciplinary collaboration created a more holistic conversation, as it combined medical issues with existential considerations related to the patients everyday life and what mattered most to them and their caregiver. Despite our finding of equal collaboration, profession was an important factor delineating the focus and roles of the nurses and physicians during the conversation. Nurses primarily addressed holistic and everyday life issues, while physicians concentrated on medical issues and treatment plans, which influenced their roles within the interdisciplinary collaboration.

Serious illness conversations are situated within palliative care, as they focus on discussions about patient’s prognosis, medical understanding, and the influence of their physical and mental condition on their values, goals, fears, and future care preferences [23]. Palliative care, as defined by The World Health Organization, adopts a holistic approach that addresses not only the patient’s physical and mental symptoms but also their social and existential circumstances [32]. Conversations involving existential conditions center around topics such as illness, anxiety, grief, and are typically conducted using an existential language rather than a descriptive language [33]. Existential language not only builds on information or facts (descriptive language), but is about subjective feelings, perceptions, and thoughts [33]. This study found that the presence of nurses in the conversation facilitated a shift in focus for patients and caregivers towards more existential issues. Likewise, the nurses’ contribution to the interdisciplinary collaboration included helping patients and caregivers express their feelings using a more existential language, which differed from the physicians’ use of descriptive language to inform about blood samples and treatment plans. The findings, therefore suggest that interprofessional collaboration during serious illness conversations can promote a holistic and existential approach, while also strengthening the integration of palliative care within the context of hematological disease trajectories. The use of both existential and descriptive language within the same conversation reflects the interdisciplinary interaction, which strengthens each professionals’ expertise and improves the quality of patient care.

Previous studies have highlighted that interdisciplinary collaboration can often be perceived as disorganized and lead to confusion regarding role responsibility [6, 19]. To address this issue,, the HCPs in this study underwent an interdisciplinary training session focusing on interdisciplinary collaboration and role responsibility. By receiving this training and engaging in a brief interdisciplinary preparation before each conversation, the HCPs experienced and improved sense of role responsibility and a more structured conversation. Consequently, the occurrence of fragmented and disorganized collaboration was avoided.

The relationship between the HCPs in the specific department and their perceptions of their own contributions may be of great importance to fully understand the impact on interdisciplinary collaboration in patient care. HCPs continually respond to one another and adapt their actions to align with what seems acceptable within their organization and culture context [34]. However, this adaptation to organizational culture can limit spontaneity during conversation, and in extreme cases, hinder conversations on topics that are important for the patient and caregiver [34]. This study contributes with new insights into the collaboration and contributions of nurses and physicians in interdisciplinary communication during serious illness conversations. Additionally, the interdisciplinary training session provided an opportunity for physicians and nurses to jointly strengthen their communication and relational skills when engaging in serious illness conversations. These findings highlight the importance of training the competencies of communication within interdisciplinary teams [34].

### Recommendation for interdisciplinary practice

Based on the findings of this study, it is recommended to implement interdisciplinary communication training and interdisciplinary collaboration dialogues before, and after serious illness conversations as it helps the nurse and physician to be focused during the conversation. This study suggests that collaboration between HCPs facilitates the use of both existential and descriptive language in conversations, allowing for comprehensive conversations on both medical issues and the patients’ existential concerns. However, to prevent fragmented and uncoordinated collaboration, it is crucial to implement organizational changes that support continuity in patient-HCP relationships. This can be achieved by providing interdisciplinary training and allocating a few minutes for preparation before and after each conversation. Based on the study’s results, we recommend conducting consultations that focus on values, hopes, fears, and preferences of both patients and caregivers in regard to future care through interdisciplinary collaboration between nurses and physicians. While it may not be common practice in many departments to offer interdisciplinary consultations for standard medical care, incorporating the presence of both a nurse and physician can be beneficial. However, considering time restraints and the high turnover rates of nurses, it is advisable to prioritize interdisciplinary consultations specifically for conversations concerning existential issues, prognostic understanding, and preferences for future care. Implementing these recommendations, healthcare organizations can increase the quality of patient care, promote effective communication, and ensure that patients holistic needs are addressed.

### Study limitations

The study had several limitations to consider. Firstly, the sampling of eligible HCP was limited to those who had previously participated in a training day and had experience with serious illness conversations. This selection process may have introduced bias and may not fully represent the broader population of HCPs. The age and seniority of the nurses in the study could have influenced their role and the dynamics of interdisciplinary collaboration. Previous research indicates that younger and less experienced nurses may be more susceptible to the influence of physicians’ behavior compared to older and more experienced nurses [35]. Secondly, the demographics were limited, with only one male HCP participant, and a majority of male patients, and female caregivers. Most of the eligible patient and caregiver dyads were spouses. It is important to consider that a more heterogenous sample could yield different insights into interdisciplinary collaboration during serious illness conversations. Secondly, all participants were from western cultures, which limits the transferability of the findings to other cultural backgrounds. Additionally, the study was conducted solely in one hematological setting, which further limits the transferability of the findings across departments, settings, hospitals, and regions. Thirdly, the study only investigates the interprofessional collaboration of nurses and physicians, but the interdisciplinary collaboration in serious illness conversations may vary within other healthcare professions.

Fourth, the small sample size of the focus groups is another limitation. While the small group size resulted in in-depth individual descriptions and natural interactions, it may limit the transferability of the findings to a larger population. Lastly, the researchers who conducted the interviews also served as trainers for the serious illness conversations. This dual role may potentially introduce interview bias and influence the responses provided by the participants. Due to the potential involvement of nurses and physicians in both the adaption process of the conversations and in this evaluation, it is important to acknowledge the potential for positive bias in the findings. The participants may have been more motivated for the conversations and feel an enhanced ownership and thereby be more positive in the evaluation. Nevertheless, we comprehend the involvement in the adaptation as a part of the implementation process.

Future research should address these limitations by including larger and more diverse samples that include a range of HCPs. Investigating interdisciplinary collaboration in serious illness conversations across various departments, hospitals, and regions would provide a more comprehensive understanding of the relationship dynamics between HCPs in different work cultures. Further, longitudinal studies can explore the development of collaboration and roles over time, and on how collaboration strengthens with increased experience in conducting these conversations.

## Conclusion

In conclusion, this study emphasizes the importance of prior relationships between HCPs and patients and caregivers in influencing interdisciplinary collaboration and the integration of nursing and medical perspectives in serious illness conversations. The study underscores the benefits of interdisciplinarity, which enable nurses and physicians to incorporate both existential and descriptive language when discussing medical and existential issues. This facilitates the active involvement of each profession in working towards the common goal of delivering optimal care to the patient and caregiver.

## Data Availability

The datasets used and/or analyzed during the current study are available from the corresponding author on reasonable request.

## List of abbreviations

MM: Multiple Myeloma
HCP: Health care professional
SICP: The Serious Illness Care Program
